# Evaluation of meaningful change in bowel movement frequency for patients with carcinoid syndrome

**DOI:** 10.1186/s41687-019-0153-y

**Published:** 2019-10-26

**Authors:** Stacie Hudgens, John Ramage, Matthew Kulke, Emily Bergsland, Lowell Anthony, Martyn Caplin, Kjell Öberg, Marianne Pavel, Jonathon Gable, Phillip Banks, Qi Melissa Yang, Pablo Lapuerta

**Affiliations:** 1 CEO & Strategic Lead, Quantitative Science, Clinical Outcomes Solutions, 1790 E. River Rd, Suite 205, Tucson, AZ 85718 USA; 2 Hampshire Hospitals NHS Foundation Trust, Aldermaston Road, Basingstoke, Hampshire, RG24 9NA UK; 3 Dana-Farber Cancer Institute, 450 Brookline Ave, Boston, MA 02215 USA; 4 UCSF Helen Diller Family Comprehensive Cancer Center, 1450 3rd St, San Francisco, CA 94158 USA; 5 University of Kentucky, 410 Administration Dr, Lexington, KY 40508 USA; 6 Royal Free Hospital, Pond St, Hampstead, London, NW3 2QG UK; 7 Uppsala University, 752 36 Uppsala, Sweden; 8 Friedrich Alexander University Erlangen-Nürnberg, Schloßplatz 4, 91054 Erlangen, Germany; 9 Lexicon Pharmaceuticals Inc., 8800 Technology Forest Pl, The Woodlands, TX USA

**Keywords:** Carcinoid syndrome, Bowel movement frequency, Meaningful change

## Abstract

**Background:**

Carcinoid syndrome is associated with a reduced quality of life that can be attributed to symptoms such as diarrhea and fatigue as well as social and financial issues. This study was conducted to psychometrically assess meaningful change in bowel movement frequency among carcinoid syndrome patients using data from the TELESTAR clinical study.

**Methods:**

An anchor-based approach for deriving meaningful change thresholds consisted of mapping change from baseline bowel movement frequency to other patient-reported assessments of change. These included the European Organization for Research and Treatment of Cancer (EORTC) Quality of Life Questionnaire - Core Questionnaire (QLQ-C30) Diarrhea Symptom responders, the EORTC Gastrointestinal NET questionnaire (GI.NET21) GI Symptom responders, and reported adequate relief at Week 12 (≥ 10-point score decrease from Day 1 to Week 12). Parameters included within-group mean change from baseline to Week 12, t-tests of the change (Wilcoxon rank sum for adequate relief), and effect size.

**Results:**

There were 135 carcinoid syndrome patients with a mean baseline frequency of 5.7 bowel movements a day. A distribution-based method yielded meaningful change estimates of 0.62 bowel movements a day for overall frequency and 0.83 bowel movements a day at Week 12. Anchor-based analysis indicated a large effect size among patients who reported adequate relief at Week 12 (− 1.58; *n* = 18; *P* = 0.014), the QLQ-C30 Diarrhea domain responders (− 1.24; *n* = 40; *P* < 0.001), and the GI.NET21 GI Symptoms Domain responders (− 1.49; *n* = 25; *P* = 0.005). Exit interview data for meaningful change yielded effect size estimates of − 1.57 for overall change during the Double-blind Treatment Period and − 1.97 for change between Baseline and Week 12.

**Conclusions:**

Meaningful change derivation is critical to interpret patient outcomes for evaluating treatment efficacy. In this study, carcinoid syndrome patients experienced clinically meaningful reductions in bowel movement frequency of ≥30% over 12 weeks with telotristat ethyl treatment.

**Trial registration:**

NCT01677910.

## Introduction

Carcinoid tumors are well-differentiated neuroendocrine tumors (NETs) that originate in neuroendocrine cells [1]. Advanced disease is associated with a reduced health-related quality of life (HRQoL) related to diarrhea, fatigue, and flushing symptoms, besides family, social, and financial issues [2–5]. These NETs usually occur in the small intestine and represent about 0.5% of all newly diagnosed malignancies with an annual incidence of approximately 2 per 100,000 persons [6, 7]. The overall 5-year survival rate is 67.2% [6]. Carcinoid syndrome (CS) is caused by bioactive compounds released into the circulation, occurring in almost 20% of NETs patients [8]. Large amounts of serotonin (5-hydroxytryptamine [5-HT]) release is believed to cause diarrhea, endocardial and mesenteric fibrosis [9–11].

The TELESTAR study was conducted to psychometrically assess meaningful change in bowel movement (BM) frequency and evaluate the efficacy and safety of telotristat ethyl in CS patients with diarrhea not adequately controlled by somatostatin analogs (SSA) (TELESTAR study; NCT01677910). The primary objective of TELESTAR was to confirm that at least 1 or more doses of telotristat ethyl was effective in reducing the number of daily BMs from baseline averaged over the 12-week double-blind period (Treatment Period) [12].

## Methods

### Study design

The design and results of TELESTAR have been previously described [13]. Briefly, patients entered a Screening/Run-in Period of 3- or 4-weeks to establish Baseline symptoms. They were then randomly assigned (1:1:1) on Day 1 to receive one of two dose levels of telotristat ethyl (250 or 500 mg) or placebo thrice daily for 12 weeks. All patients remained on their baseline dose of SSA therapy during the Treatment Period. Subsequently, they participated in a 36-week Open-label Extension Period when everyone received 500 mg of the active study drug thrice daily. This study received Institutional Review Board approval.

The focus is on the primary endpoint, change from Baseline in BM frequency during the Double-blind Treatment Period. The intent-to-treat (ITT) analysis population included all randomized patients. All analyses populations were derived from the ITT dataset. All patients participating in the patient interview substudy after the Double-blind Treatment Period were included in the patient interview subpopulation (ISP).

### Study instruments

Evaluation of meaningful change in BM frequency required the inclusion of other supportive clinical outcomes assessments: a Yes/No question about CS gastrointestinal symptom relief; European Organization for Research and Treatment of Cancer (EORTC) Quality of Life Questionnaire - Core Questionnaire (QLQ-C30) and EORTC Gastrointestinal NET questionnaire (GI.NET21) scales; and patient exit interview responses.

#### Number of daily BMs

Patients electronically recorded the number of daily BMs. The average BM number was mapped to individual analyses using the following criteria: difference between average Baseline BM frequency and the overall average BM frequency; and the difference between average Baseline BM frequency and average BM frequency at Week 12.

#### EORTC QLQ-C30 and GI.NET21

The QLQ-C30 contained 30 questions incorporated into 5 functional domains (Physical, Role, Cognitive, Emotional, and Social), 9 symptom scales (Fatigue, Pain, Nausea and Vomiting, Dyspnea, Insomnia, Appetite Loss, Constipation, Diarrhea, and Financial Difficulties), and a single global HRQoL/Global Health Status score [14].

The GI.NET21 module contained 21 questions: 4 single-item assessments about muscle and/or bone pain, body image, information, and sexual functioning, plus 17 items organized into 5 scales: Endocrine Symptoms (3 items), GI Symptoms (5 items), Treatment-related Symptoms (3 items), Social Functioning (3 items) and Disease-related Worries (3 items) [15].

#### Exit interviews

English- and German-speaking patients were invited to participate in the exit interview study as prespecified in the TELESTAR protocol [16]. All participants consented to the interview procedure to be conducted within 2 weeks after they completed the 12-week Double-blind Treatment Period or early termination. Patients were categorized based on reported satisfaction with improvement over the course of treatment (“very satisfied”; “somewhat satisfied”; “neither satisfied nor dissatisfied”; “somewhat dissatisfied”; or “very dissatisfied”). Patients were also categorized according to perception of BM frequency reduction (“a great deal better”; “much better”; “a little better”; “the same”; “a little worse”; “much worse”; or “a great deal worse”).

### Analytic methods

Analyses focused on the derivation and evaluation of thresholds to interpret meaningful change and responsiveness in BM frequency. All patients were included irrespective of receiving treatment (*n* = 90) or placebo (*n* = 45). Analyses was conducted using SAS Version 9.3 or higher (SAS Institute, Cary, NC, USA) [17].

Meaningful change on a patient-centered endpoint referred to the smallest difference in scores in the domain of interest (e.g., symptom or functional score), which patients perceived as beneficial. This could then be used further to discriminate between treatment groups and develop a thorough understanding of the HRQOL impact of BM frequency reduction [18, 19].

Change in BM frequency from Baseline was used to develop two individual distribution-based estimates: (1) overall change from Baseline, defined as the difference between average BM frequency during the Run-In Period and average BM frequency during the Double-Blind Treatment Period; and (2) change from Baseline at Week 12, defined as the difference between average BM frequency during the Run-In Period and 7-day average BM frequency at Week 12. Distribution-based thresholds were derived for both estimates independently using the ½ standard deviation (SD) rule [18], which is ½ the SD of both estimates.

The anchor-based approach to derive meaningful change thresholds consisted of mapping change from Baseline in BM frequency to other patient reported assessments of change. The relationship between BM frequency and each continuous patient-reported outcome (PRO) anchor was evaluated prior to inclusion in the anchor based analysis using correlational analyses. The criterion threshold value for determining if the anchor was correlated with the outcome is a correlation coefficient > 0.30 at Baseline, Week 12, or change from Baseline [18]. Anchor-based thresholds were developed by calculating mean change and the associated effect size (ES) statistic for each anchor-based. The ES was calculated from the difference between average score in BM frequency over 12 weeks and average Baseline BM frequency, this difference being divided by the SD of average Baseline BM frequency. A negative ES represented BM frequency reduction compared to Baseline [19, 20]. An additional analysis was conducted, where ES was calculated as the mean score in average BM frequency at Week 12 minus the average Baseline BM frequency divided by the SD of the group average Baseline BM frequency. For both analyses, a single value (or range of values for interpreting change in BM frequency) was developed for the full ITT population by selecting the mean improvement for each analytic group. These thresholds could be applied to stratify patients within each treatment arm by mean change in BM frequency relative to the identified threshold. Negative values of ES indicated reductions from Baseline in BM frequency.

Responsiveness refers to the ability of an assessment to detect change where it exists [18]. To assess responsiveness, patients were defined as improved or worsened based on meaningful change in prespecified categorical endpoints. An absolute improvement of 10 points from Day 1 to Week 12 defined improvement in each of the EORTC domains [20]. The analysis of covariance (ANCOVA) procedure was used to calculate the *P*-value and adjusted for age, sex, and race. The within-group level of change in individual scores was expressed as a standardized effect size (SES), calculated as the mean change score between Baseline and Week 12, and divided by the SD of the pooled population at Baseline. Based on Cohen’s recommendations, the following values represent the magnitudes of responsiveness: small change (SES = 0.20), moderate change (SES = 0.50), and large change (SES = 0.80) [21]. Statistically significant differences in change in scores between groups were tested through an ANCOVA model adjusting for age, sex, and race.

To demonstrate the application of MCT in responsiveness evaluation, unblinded cumulative distribution function (CDF) curves are presented, calculated as the cumulative percentage of patients achieving various threshold levels of change from Baseline in either the overall average daily BMs or the average BMs during Week 12 by treatment arm.

## Results

A total of 135 patients were included in the ITT population at baseline (Run-in Period), Day 1, and 120 patients were included at Week 12. Baseline characteristics were generally similar across all treatment groups [13]. The numbers of patients with available PRO data at Week 12 by parameter were: *n* = 108 (90.0%) average daily BM; *n* = 114 (95.0%) adequate relief; *n* = 113 (94.2%) EORTC QLQ-C30; and n = 113 (94.2%) GI.NET21 data.

### Interpretation of scores: meaningful change

#### Relationship between potential PRO anchors and BM frequency

Only the QLQ-C30 Diarrhea symptom score yielded a correlation coefficient with average BM frequency exceeding the acceptability threshold of > 0.30 at Week 12 (Pearson = 0.42; Spearman = 0.45). Correlation was at least equal to the acceptability threshold for two items: (1) between change from baseline in the QLQ-C30 Diarrhea symptom score at Week 12 and in overall BM frequency (Pearson = 0.38; Spearman = 0.31); (2) and a correlation between change from baseline in GI.NET21 GI Symptoms domain score at Week 12 and change from baseline in overall BM frequency (Pearson = 0.30; Spearman = 0.30).

#### Anchor-based analysis of meaningful overall change from baseline in BM frequency

An anchor-based analysis of thresholds for meaningful overall change in BM frequency produced a large ES (defined as > 0.80) among ITT patients reporting adequate relief at Week 12 (− 1.20), EORTC QLQ-C30 Diarrhea Symptom scale (− 0.83) and EORTC GI.NET21 GI Symptoms scale (− 1.25) responders. Wilcoxon Rank Sum test analysis indicated a significant difference in adequate relief groups (*P*-value = 0.011), EORTC QLQ-C30 Diarrhea Symptoms domain (*P*-value = 0.019), and EORTC GI.NET21 GI Symptoms domain responders and non-responders (*P*-value < 0.001) (Table 1).

**Table 1:** Summary of Meaningful Change Thresholds

Parameter	Threshold for Acceptability	Analysis Population	Average Number of Daily BMs					
			Overall Change from Baseline			Change from Baseline at Week 12		
			N	ES	Mean	N	ES	Mean
Anchor-based Meaningful Change Thresholds								
Adequate relief (changed to Yes at Week 12)	Effect Size: -Small (≥ 0.2) -Moderate (≥ 0.5) -Large (≥ 0.8)	ITT	19	−1.20	−1.90	18	−1.58	−2.52
EORTC QLQ-C30 Diarrhea (Responder)		ITT	45	−0.83	−1.71	40	−1.24	−2.56
EORTC GI.NET21 GI Symptoms (Responder)		ITT	30	−1.25	−1.88	25	−1.49	−2.27
Distribution-based Meaningful Change Thresholds								
−1/2 Baseline SD	N/A	ITT	135	N/A	0.62	108	N/A	0.86

*BM* Bowel movement, *EORTC QLQ-C30* European Organization for Research and Treatment of Cancer Quality of Life Questionnaire - Core Questionnaire, *ES* Effect size, *ITT* Intent-to-treat, *N/A* Not applicable, *SD* Standard deviation

**Fig. 1:**
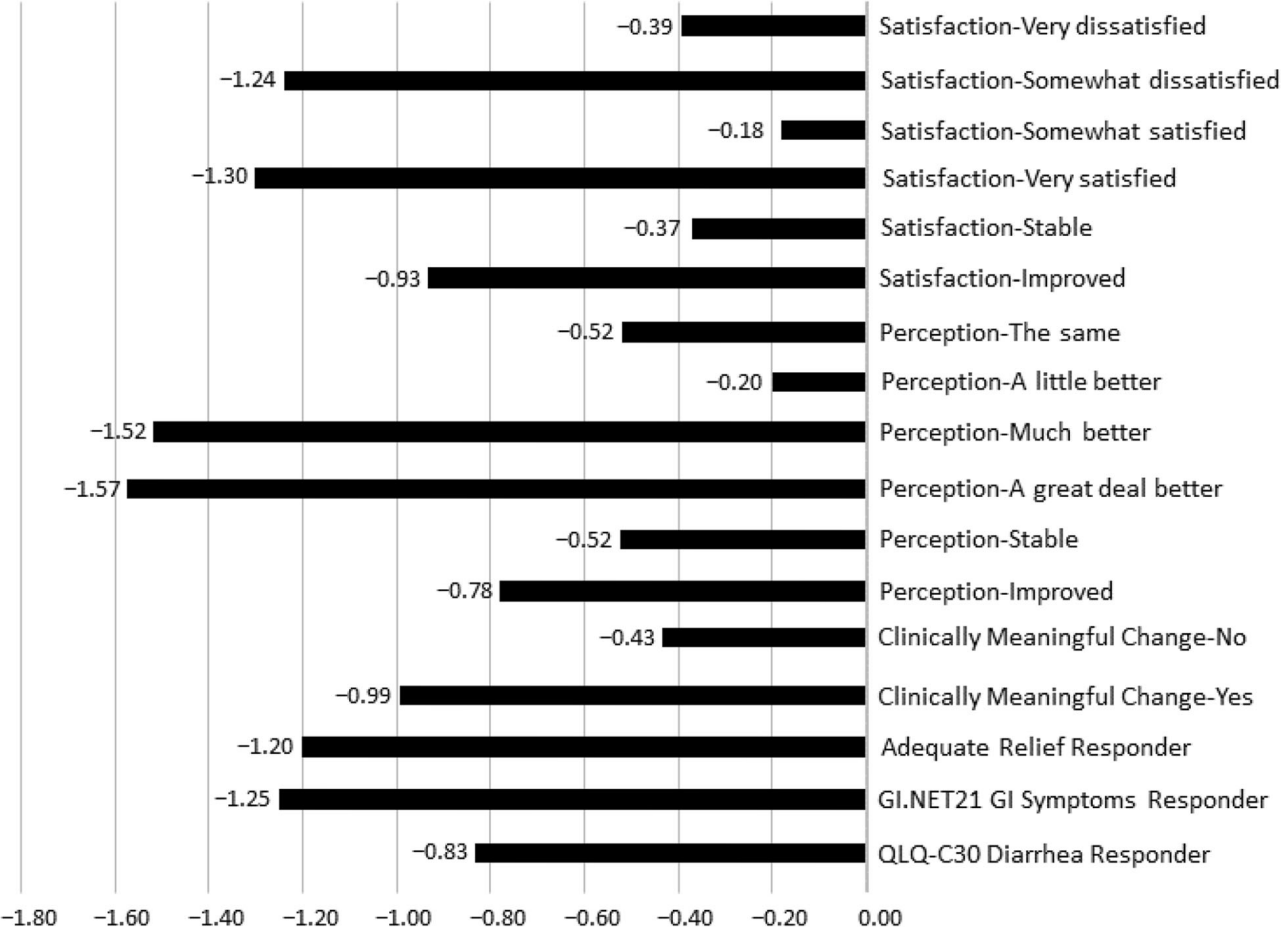
Effect Sizes of Change in Overall BM Frequency from Baseline (ISP population)

Large ES estimates were observed among ISP patients whose perception of change was “a great deal better” (− 1.57) and “much better” (− 1.52), patients who reported being “very satisfied” (− 1.30) and “somewhat dissatisfied” (− 1.24) with symptom relief, the collapsed improvement category for carcinoid symptom relief (− 0.93), and those that considered their perception of change in BM frequency to be clinically meaningful (− 0.99). Kruskal-Wallis Exact test indicated a significant difference between the perceptions of change in BMs response groups (*P*-value = 0.005) (Fig. 1).

#### Analysis of meaningful change in BM frequency at week 12

Anchor-based analysis indicated a large ES among ITT patients who reported adequate relief at Week 12 (− 1.58), responders on the EORTC QLQ-C30 Diarrhea domain (− 1.24), and the EORTC GI.NET21 GI Symptoms Domain (− 1.49) (Table 1). Wilcoxon Rank Sum test analysis indicated a significant difference in adequate relief groups (*P*-value = 0.014), EORTC QLQ-C30 Diarrhea Symptoms domain (*P*-value < 0.001), and EORTC GI.NET21 GI Symptoms domain responders and non-responders (*P*-value = 0.005).

**Fig. 2:**
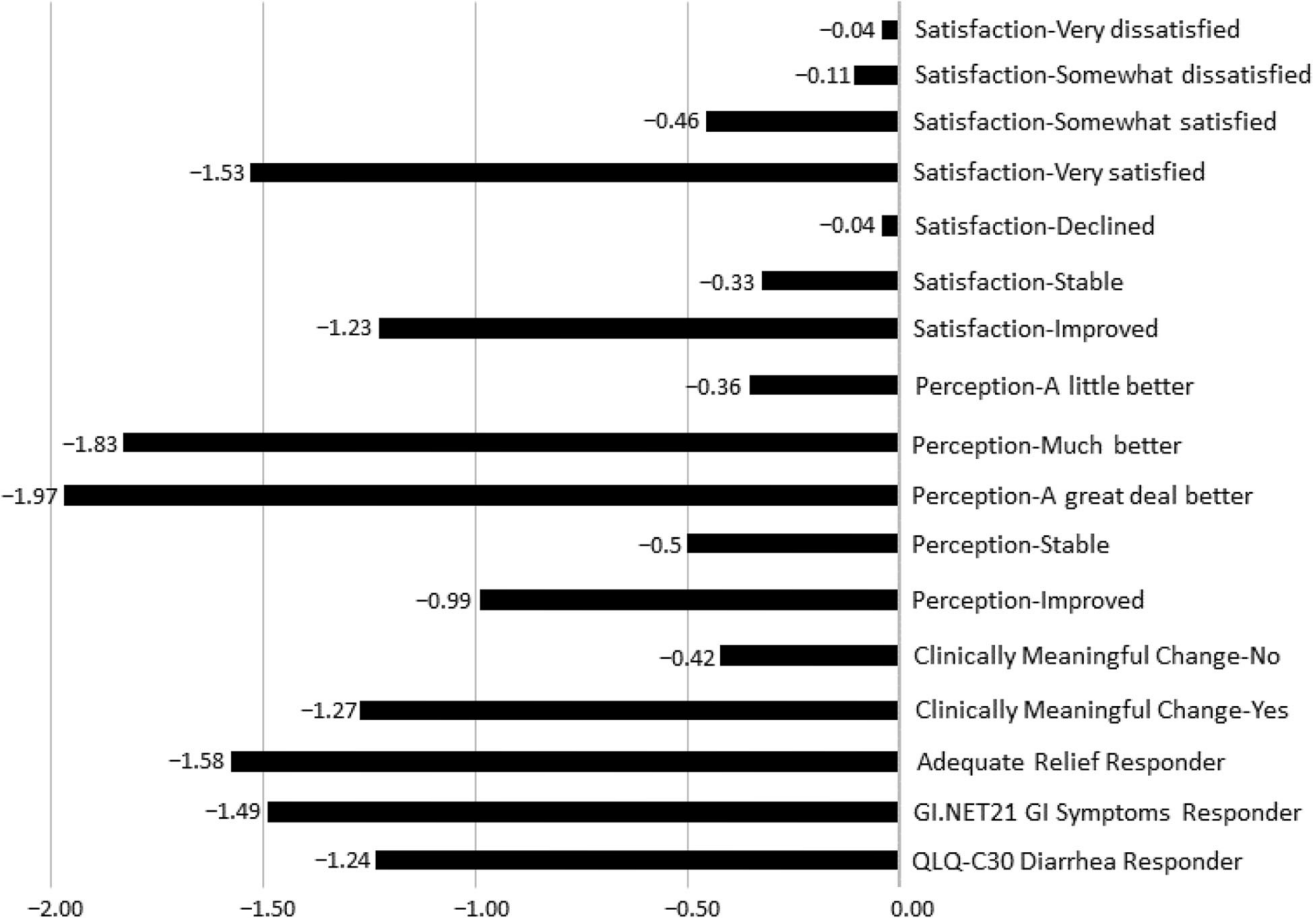
Effect Sizes of Change in BM Frequency at Week 12 from Baseline (ISP population)

A large ES was observed among ISP patients who reported change of “A great deal better” and “Much better” in BMs (− 1.97, − 1.83, respectively), patients who improved in the collapsed perception categories (− 0.99), patients who reported being “very satisfied” with the relief (− 1.53), improved in the collapsed satisfaction categories (− 1.23), and patients who reported their perception of change in BM frequency clinically meaningful (− 1.27) (Fig. 2). Kruskal-Wallis Exact test indicated a significant difference between the perceptions of change in BM response groups (*P*-value = 0.029).

#### Distribution-based thresholds

Overall, the thresholds for meaningful change in overall average BM frequency at baseline and the change in average BM frequency from baseline at Week 12 were 0.62 and 0.86 BM/day, respectively (Table 1).

#### Responsiveness

Responsiveness analysis suggested that change from baseline in overall average BM frequency was moderately responsive to patient reported changes (Table 2). There were large reductions in average weekly BMs among patients indicating a change from “No” at baseline to “Yes” in adequate relief at Week 12 (SES = − 0.99), patients that indicated a great deal of improvement in BMs (SES = − 1.44), and among patients who were “Very satisfied” (SES = − 1.18) with their relief. The number of ISP patients stratified into the perception of change and satisfaction with study medication response was sub-optimal.

**Table 2:** Summary of Responsiveness Results

Parameter	Threshold for Acceptability	Analysis Population	Average Daily BMs			
			Overall Change from Baseline		Change from Baseline at Week 12	
			N	SES	N	SES
Responsiveness (SES)						
Adequate relief (changed to Yes at Week 12)^a^	Standard Effect Size: -Small (≥ 0.2) -Moderate (≥ 0.5) -Large (≥ 0.8)	ITT	19	−0.99	18	−1.40
Perception of change in BMs^b^						
A great deal better		ISP	9	−1.44	8	−1.80
Much better		ISP	4	−0.50	4	−0.60
Satisfaction with study medication—Relief of CS^c^						
Very satisfied		ISP	12	−1.18	12	−1.38
Somewhat satisfied		ISP	7	−0.15	6	−0.38

*BM* Bowel movement, *CS* Carcinoid syndrome, *ISP* Interview subpopulation, *ITT* Intent-to-treat, *SES* Standardized effect size

^a^There were *n* = 75 “not changed to yes at Week 12”

^b^There were *n* = 10 “a little better”; n = 10 “the same”; and n = 1 “a little worse”

^c^There were *n* = 8 “neither satisfied nor dissatisfied”; *n* = 3 “somewhat dissatisfied”; and n = 3 “very satisfied”

Responsiveness analysis indicated slightly greater reductions in BM frequency from baseline at Week 12 when compared to change during the Double-blind Treatment Period. Large negative SES values were observed across several item response categories, including patients who changed their response on the adequate relief item to “Yes” at Week 12 as well as those that continued to report “No” (SES = − 1.40 and − 0.81, respectively), patients whose self-reported perception of change was “A great deal better” (SES = − 1.80), and among patients who reported being “Very satisfied” with their relief of CS symptoms (SES = − 1.38).

**Fig. 3:**
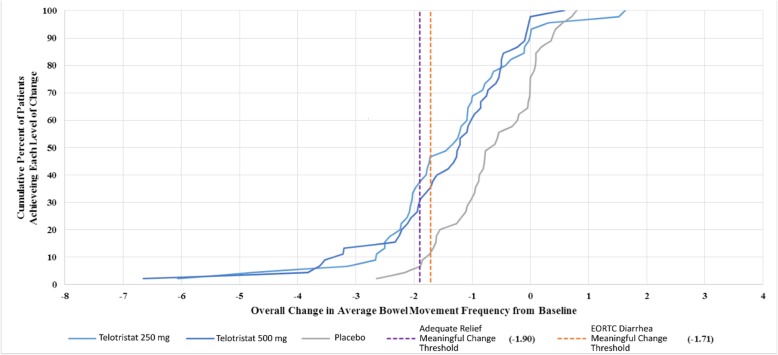
CDF of Overall Change from Baseline in Average BM Frequency

**Fig. 4:**
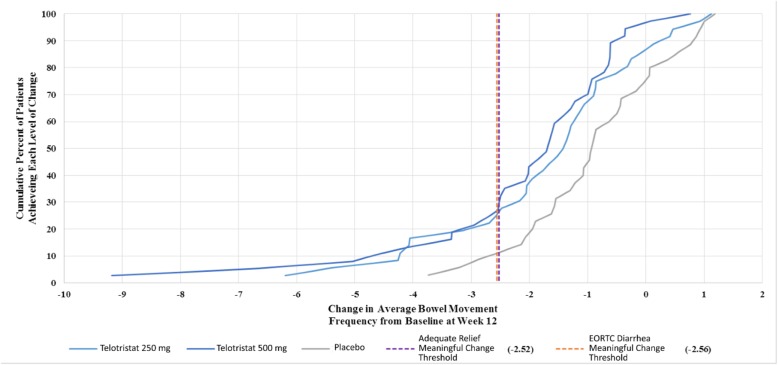
CDF of Change in Average BM Frequency from Baseline at Week 12

Cumulative distribution function curves in Figs. 3 and 4 show a clear separation of treatment and placebo at identified thresholds of meaningful change.

## Discussion

Diarrhea is a prominent feature of CS, which may require a different impact measurement approach than other forms of diarrhea to understand clinically meaningful change. A distribution-based method yielded meaningful change estimates of 0.62 BM/day for overall BM frequency and 0.83 BM/day at Week 12, indicating that patients whose average BM frequency increased or decreased by more than that level were reporting a meaningful change. An anchor-based approach was developed using exit interview data and change in scores. The QLQ-C30 Diarrhea Symptom and GI.NET21 GI Symptoms scale were found to have an association > 0.3, and were thus used to derive estimates of meaningful change. Specifically, change from baseline in the QLQ-C30 Diarrhea symptom score at Week 12 correlated with Change from baseline in Overall BM frequency at 0.38. When applying exit interview data as the basis for meaningful change, patients’ perception of change in BMs since beginning the study yielded ES estimates of − 1.57 for overall change during the Double-blind Treatment Period and − 1.97 for change between baseline and Week 12.

When analyzing the responsiveness of BM frequency to various levels of patient reported change, ES estimates for the perception of change item for both overall change and change from Week 12 were significantly different despite the low subject numbers interviewed after the Double-blind Treatment Period. The ES estimates (and the accompanying threshold of − 1.97 BMs) could be valuable when approximating meaningful change in BM frequency. When applying these thresholds to the clinical change results in the CDF curves, the trend towards greater reductions in BM frequency among the treatment patients indicate a higher percentage would exceed the anchor and distribution-based thresholds, although this would need to be confirmed as part of an efficacy analysis.

Current clinical outcome assessment research has demonstrated that it is possible to establish defensible responder thresholds, which support informed treatment decisions [22]. Some physicians interpret clinically meaningful change based on means: however, it is important to examine the distribution of response as in the CDF. The relevance of the CDF is reflected by its inclusion in product labeling [23]. Figure 4 shows that some responders achieved reductions in the range of 3 to 9 BMs/day on treatment, but not on placebo. These are results averaged over 12-weeks, corresponding to BM reductions during this time frame.

A limitation is that a minimum change is difficult to identify. The mean BM frequency change in a responder group is not necessarily a minimum; it would be of interest to examine empiric density distributions to see which levels of BM frequency change distinguish between responders and non-responders.

## Conclusions

Meaningful change derivation is critical for the interpretation of patient outcomes in the evaluation of treatment efficacy. Analyses identified a meaningful change in BM frequency which would coincide with improvement across each of the patient reported anchors (retrospective and prospective). More specifically, CS patients treated with telotristat ethyl experienced clinically meaningful reductions in BM frequency of ≥30% over 12 weeks.

## Data Availability

The datasets generated and/or analyzed during the current study are not publicly available due to confidentiality but are available from the corresponding author on reasonable request.

## Abbreviations

ANCOVA: Analysis Of Covariance
BM: Bowel movement
CDF: Cumulative distribution function
CS: Carcinoid syndrome
EORTC: European Organization for Research and Treatment of Cancer
ES: Effect size
GI.NET21: EORTC Gastrointestinal NET
HRQoL: Health-related quality of life
ISP: Interview subpopulation
ITT: Intent-to-treat
NET: Neuroendocrine tumors
PRO: Patient-reported outcome
QLQ-C30: Quality of Life Questionnaire - Core Questionnaire
SD: Standard deviation
SES: Standardized effect size
SSA: Somatostatin analogs
